# Mapping the psychosocial network of Kenyan adolescents: the pivotal role of loneliness and gender-specific pathways

**DOI:** 10.3389/fpubh.2025.1642144

**Published:** 2025-10-30

**Authors:** Fei-Rui Ni, Zhen-Xing Huang, Yun Chen

**Affiliations:** Wenzhou Seventh People's Hospital, Wenzhou, China

**Keywords:** loneliness, adolescent, mental health, network analysis, gender differences

## Abstract

**Background:**

Loneliness is increasingly recognized as a critical yet understudied determinant of adolescent mental health, particularly in low- and middle-income countries. While its prevalence and impact have been well-documented in Western contexts, little is known about its role within the psychosocial networks of youth in many African contexts, where social structures and gender norms may diverge sharply.

**Methods:**

We conducted a cross-sectional network analysis using data from 1,445 Kenyan secondary school students. Participants completed validated self-report measures of depression, anxiety, loneliness, social support, optimism, happiness, gratitude, and key demographic variables. Mixed graphical models were employed to examine the global structure and centrality of variables within the adolescent psychosocial network. Gender-stratified analyses and network comparison tests were used to identify sex-specific differences in network architecture and key pathways.

**Results:**

Loneliness emerged as the most central psychological variable, directly bridging depressive symptoms, diminished wellbeing, and social support. Gender-stratified networks revealed notable divergences: the positive association between depression and loneliness was significant for girls (edge weight = 0.15) but was not significant appeared in the male network (a statistically significant difference, *p* = 0.040), while peer support more strongly buffered loneliness for boys. Furthermore, family support was more central for girls, whereas support from friends was more central for boys.

**Conclusions:**

These findings highlight loneliness as a pivotal and gender-contingent node within adolescent psychosocial networks in Kenya. Network-based approaches reveal unique pathways of distress and resilience, underscoring the need for contextually and gender-sensitive interventions.

## 1 Introduction

Loneliness has rapidly emerged as one of the most pressing—and yet less understood—public health challenges facing adolescents worldwide. Often described as a “silent epidemic” (1), loneliness is now recognized not only as a ubiquitous subjective experience but as a powerful determinant of psychological and physical health across the lifespan (2, 3). Among young people, the prevalence of loneliness has soared in recent years, exacerbated by shifting social dynamics, digital connectivity, and, in many regions, persistent socioeconomic instability (4). While loneliness is frequently linked to adverse mental health outcomes such as depression and anxiety, emerging research suggests it is far more than a symptom or byproduct: it is a central and potentially causal node in the architecture of adolescent psychological distress (5).

The urgency of understanding the dynamics of loneliness is even greater in low- and middle-income countries, where adolescents often face unique constellations of risk—ranging from social exclusion and academic pressure to economic precarity and rapid urbanization (6). In sub-Saharan Africa, youth populations are expanding faster than anywhere else on the globe, yet their psychosocial experiences remain profoundly understudied. Addressing loneliness in these contexts is not only a scientific imperative but a public health priority, given its catalytic role in shaping trajectories of mental health, wellbeing, and life chances. Despite this, the centrality of loneliness in adolescent mental health—its mechanisms, correlates, and contextual expressions—remains poorly mapped, especially in African settings where traditional protective structures are under strain and gendered experiences may diverge sharply (7).

Although loneliness is universally experienced, its prevalence and psychological correlates are shaped by cultural, socioeconomic, and developmental factors (8), making it imperative to understand its role within diverse contexts such as Kenyan secondary schools. Recent theoretical advances and empirical findings suggest that loneliness acts not merely as a symptom or byproduct of psychopathology, but as a central node within the broader psychosocial network, bridging negative affect, diminished social support, and reduced positive psychological resources (9). Yet, the interplay between loneliness and other determinants of adolescent mental health—including perceived social support, optimism, gratitude, happiness, and perceived academic control—remains under-explored in African settings, where social and familial structures may operate differently from Western norms.

Existing research has established the protective effects of social support and positive psychological variables in mitigating adolescent distress (10). However, most studies have examined these factors in isolation or via linear, variable-centered approaches, which obscure the complex, interdependent relationships among multiple psychosocial domains (11). Network analysis, a rapidly evolving methodological paradigm, offers a powerful lens to map and quantify these associations, revealing how central symptoms or protective factors may propagate their influence throughout the mental health ecosystem (12, 13). Despite its potential, network approaches have rarely been harnessed to elucidate the unique psychosocial architectures of African adolescents, and virtually no studies have systematically examined how these network structures may differ by gender—a critical omission, given the robust evidence for gender disparities in the prevalence, expression, and correlates of adolescent mental health problems (14).

Gender differences are particularly salient in the context of loneliness. Globally, adolescent girls tend to report higher levels of internalizing symptoms and are often more sensitive to relational stressors, whereas boys may derive greater benefit from peer-based support (15). In Kenyan settings, evidence on gendered patterns of psychological distress, social support, and positive psychological characteristics is emergent but suggests that cultural and structural factors may amplify or attenuate these differences (16). Yet, the precise network pathways linking loneliness, depression, anxiety, social support, and positive traits—and how these pathways diverge by gender—remain poorly understood.

Critically, few studies to date have leveraged large, representative samples of African adolescents to (a) map the centrality and bridging role of loneliness within the psychosocial network, (b) quantify the multivariate associations among mental health symptoms, positive psychological resources, and demographic factors, and (c) test for systematic gender differences in network structure and key pathways. This gap limits the field's capacity to design targeted, contextually appropriate interventions that address the most influential nodes and connections within adolescent psychosocial systems.

The value of network analysis in this context has been demonstrated in a foundational study (17), which utilized the same dataset to explore the network associations between loneliness, depression, and anxiety symptoms. Their work established loneliness as a significant node connected to internalizing symptoms in Kenyan youth. However, their analysis was primarily focused on this triad of psychopathology. The broader psychosocial ecosystem—encompassing protective factors such as optimism, gratitude, and happiness, as well as different sources of social support—remains unmapped. Furthermore, while socio-cultural factors were considered, a systematic and statistically rigorous comparison of the network structures between genders has not been conducted.

The present study addresses these limitations by employing state-of-the-art network modeling techniques in a large, diverse sample of Kenyan secondary school students. Specifically, we aim to: (1) elucidate the global network structure linking depression, anxiety, loneliness, social support, optimism, happiness, gratitude, academic control, and demographic variables; (2) identify the centrality and bridging roles of loneliness within this network; and (3) systematically examine gender differences in network structure, with a focus on the neighborhood and impact of loneliness. We hypothesize that loneliness will emerge as a central node bridging psychological distress and protective factors, and that network pathways involving loneliness and social support will differ significantly between boys and girls.

## 2 Method

### 2.1 Participants and data collection

This study conducted a secondary analysis of publicly available data from Kenyan adolescents, originally collected as baseline data from a randomized controlled trial of a positive psychology intervention (Shamiri) conducted in the greater Nairobi area of Kenya during summer 2019. The original trial was registered in the Pan African Clinical Trials Registry (PACTR201906525818462), and detailed information about the intervention can be found elsewhere (18). The dataset comprises self-reported measures from 2,192 students recruited from four secondary schools located in Nairobi and Kiambu counties, Kenya. Schools were selected by the original research team to reflect diversity in academic resources and student backgrounds, including two national boarding schools (one boys', one girls'), an all-girls day school, and a mixed-gender day school. All procedures for the original data collection were approved by the Maseno University Ethics Review Committee (MUERC) prior to the start of data collection. School principals and administrators from the four participating schools notified students about the study, and students who elected to participate joined the study team after school to complete the questionnaires. Informed consent/assent was obtained from all guardians and participants according to the original study protocol.

For the current secondary analysis, we began with the full dataset of 2,192 students and applied a systematic exclusion procedure to derive the final analytic sample. Specifically, we excluded participants outside the 13–18 age range (*n* = 58), those with missing gender data (*n* = 25), and those with substantial missingness on core psychological scales (*n* = 664), resulting in a final analytic sample of 1,445 students. To assess for potential selection bias from this process, an attrition analysis was conducted comparing the final sample (*N* = 1,445) with the excluded participants (*N* = 747) on key demographic variables. To address potential selection bias from the sample reduction (from *N* = 2,192 to *N* = 1,445), we conducted an attrition analysis comparing the final analytic sample with excluded participants (*N* = 747). Using *t*-tests and chi-squared tests, we reported both *p*-values and effect sizes (Cohen's *d* and Cramér's *V*) to assess practical importance beyond statistical significance. The analysis revealed no significant differences for most measures of social support, happiness, and academic control. While a statistically significant difference was found for gender (*p* = 0.007), the negligible effect size (Cramér's *V* = 0.059) indicates this difference is not practically meaningful. However, the analytic sample did report significantly higher levels of depression (*d* = −0.20), anxiety (*d* = −0.18), optimism (*d* = −0.38), and notably, loneliness (*d* = −0.59) compared to the excluded group. These findings suggest our final sample may represent a slightly more psychologically distressed population, a common occurrence in survey research.

### 2.2 Measures

Depression was assessed using the Patient Health Questionnaire-8 (PHQ-8), an eight-item scale validated for use among Kenyan adolescents, with total scores computed by summing item responses. Anxiety was measured with the Generalized Anxiety Disorder-7 (GAD-7) scale, also validated for this population. Perceived social support was captured using the Multidimensional Scale of Perceived Social Support (MSPSS), with subscales for support from significant others, family, and friends, and a total support score. Positive psychological characteristics were measured by the EPOCH subscales for optimism and happiness, and the Gratitude Questionnaire-6 (GQ-6) for gratitude. Perceived academic control was assessed using a 6-item version of the Perceived Control Scale (PCS). Loneliness was measured using the UCLA Loneliness Scale. Additionally, demographic variables included gender, sports participation, family financial status, home location.

All scale scores were computed according to standard scoring protocols, with missing data handled via pairwise deletion within scales.

### 2.3 Network analysis

Prior to analysis, all continuous variables were standardized (*z*-scores) to ensure comparability of edge weights in the network models. Categorical variables (gender, sports participation, financial status, home location) were retained in their original coding, as appropriate for mixed graphical models.

Core study variables included loneliness, depression, anxiety, three dimensions of social support, optimism, happiness, academic control, gratitude, gender, sports participation, financial status, home location. Variables were grouped thematically as “Mental Health,” “Social Support,” “Positive Psychology,” and “Demographics” for the purposes of network visualization and interpretation. To capture the complex interrelations among a mix of continuous and categorical variables, we estimated a Mixed Graphical Model (MGM) using the estimateNetwork function from the bootnet package, specifying variable types and levels accordingly. The extended Bayesian Information Criterion (EBIC) was used for model selection (γ = 0.5), providing a conservative balance between model fit and parsimony. Custom edge coloring was employed—continuous-positive (blue), continuous-negative (red), categorical (gray)—to enhance interpretability. Network visualization was implemented with qgraph, using a spring layout and colorblind-friendly palette, with node colors reflecting variable groupings. Centrality indices (strength, closeness, betweenness, expected influence) were computed, and centrality plots generated to identify key variables within the network structure. Network stability was assessed via case-dropping bootstrapping (bootnet), with the correlation stability coefficient (CS-coefficient) evaluated for robustness.

To investigate gender differences in the network structure of adolescent mental health, we conducted parallel network analyses for female and male students, using only continuous variables to maximize comparability and statistical power. Separate Gaussian graphical models (EBICglasso) were estimated for each gender group. Edge colorings reflected the direction of associations. To ensure direct comparability, both networks were visualized with the same layout and scaling. Centrality indices were calculated for each group, and barplots were used to compare strength centrality across genders.

Network structure invariance between genders was formally tested using the Network Comparison Test (NCT) as implemented in the NetworkComparisonTest package. This permutation-based procedure assessed (a) global strength differences, (b) specific edge differences, and (c) centrality differences between male and female networks. Significance was evaluated via 100 permutations. Specific attention was given to the network neighborhood of loneliness, with edge weights compared between genders, and differences visualized via barplots and heatmaps to highlight key gender-specific predictors.

## 3 Results

### 3.1 Descriptive statistics

The analytic sample comprised 1,445 Kenyan adolescents, predominantly female (62.6%), with a balanced distribution across ages 13–18 (see Table 1). On average, the sample reported moderate levels of depression (*M* = 7.91, SD = 5.14) and anxiety (*M* = 7.42, SD = 5.14), alongside relatively high perceived social support (*M* = 5.16, SD = 1.12) and moderate-to-high levels of positive psychological variables such as optimism, happiness, and gratitude. Gender differences were evident across multiple domains. Female students reported notably higher mean depression and anxiety scores than males, suggesting a greater psychological symptom burden among girls. This pattern was consistent across both PHQ-8 (females: *M* = 8.29; males: *M* = 7.26) and GAD-7 (females: *M* = 7.73; males: *M* = 6.90), indicating that emotional distress may be more pronounced in adolescent girls within this context. In contrast, perceived social support, optimism, happiness, and gratitude showed only minimal gender differences, with both groups reporting similar levels overall. Socioeconomic variation was also reflected in the data, but gender disparities in core mental health outcomes remained robust across financial strata. For instance, even among students from wealthier backgrounds, girls exhibited higher mean depression and anxiety scores relative to boys. Sports participation was more common among males, but its association with mental health and positive psychological attributes appeared consistent across genders.

**Table 1:** Demographic characteristics and descriptive statistics of the study sample (*N* = 1,445).

**Variable**	**Level**	***N* (Percent)**	**PHQ-9**	**GAD-7**	**MSPSS**	**EPOCH optimism**	**EPOCH happiness**	**PCS academic**	**Gratitude**	**UCLA_Total**
Age		1,445 (100%)	7.91 (5.14)	7.42 (5.14)	5.16 (1.12)	3.56 (0.91)	3.36 (0.98)	2.75 (0.40)	35.43 (5.93)	19.32 (4.89)
	13	49 (3.4%)	7.53 (4.98)	7.12 (4.79)	5.03 (1.00)	3.46 (0.85)	3.40 (1.04)	2.66 (0.54)	34.41 (6.54)	19.88 (4.88)
	14	379 (26.2%)	7.01 (4.81)	6.65 (4.75)	5.23 (1.16)	3.63 (0.88)	3.49 (0.96)	2.80 (0.34)	35.87 (6.04)	18.95 (4.97)
	15	504 (34.9%)	7.58 (4.88)	7.41 (5.17)	5.14 (1.11)	3.54 (0.90)	3.32 (0.97)	2.75 (0.41)	35.66 (5.53)	19.22 (4.80)
	16	366 (25.3%)	8.77 (5.41)	7.59 (5.31)	5.20 (1.06)	3.55 (0.97)	3.35 (1.03)	2.74 (0.41)	35.32 (5.76)	19.28 (4.75)
	17	124 (8.6%)	9.21 (5.54)	9.20 (5.45)	5.06 (1.14)	3.54 (0.88)	3.24 (0.87)	2.68 (0.46)	34.23 (7.05)	20.24 (5.25)
	18	23 (1.6%)	10.13 (5.83)	8.48 (4.73)	4.77 (1.31)	3.55 (0.78)	3.09 (0.88)	2.76 (0.37)	33.57 (6.64)	21.87 (4.69)
Gender		1,445 (100%)	7.91 (5.14)	7.42 (5.14)	5.16 (1.12)	3.56 (0.91)	3.36 (0.98)	2.75 (0.40)	35.43 (5.93)	19.32 (4.89)
	Male	904 (62.6%)	8.29 (5.26)	7.73 (5.21)	5.17 (1.14)	3.59 (0.89)	3.39 (0.98)	2.74 (0.42)	35.74 (6.02)	19.74 (4.89)
	Female	541 (37.4%)	7.26 (4.86)	6.90 (4.98)	5.14 (1.07)	3.51 (0.94)	3.32 (0.98)	2.77 (0.37)	34.91 (5.76)	18.61 (4.81)
Financial Status		1,445 (100%)	7.91 (5.14)	7.42 (5.14)	5.16 (1.12)	3.56 (0.91)	3.36 (0.98)	2.75 (0.40)	35.43 (5.93)	19.32 (4.89)
	Poor	78 (5.4%)	7.23 (4.65)	6.88 (5.03)	5.21 (1.26)	3.58 (0.90)	3.57 (0.95)	2.58 (0.55)	33.86 (6.58)	17.97 (4.43)
	Not quite well-off	896 (62.0%)	7.78 (5.02)	7.23 (5.09)	5.21 (1.09)	3.54 (0.91)	3.43 (0.96)	2.77 (0.37)	35.66 (5.62)	18.94 (4.83)
	Quite well-off	408 (28.2%)	8.19 (5.38)	7.67 (5.17)	5.08 (1.12)	3.59 (0.90)	3.22 (0.99)	2.75 (0.43)	35.19 (6.29)	20.30 (4.95)
	Wealthy	63 (4.4%)	8.76 (5.58)	9.02 (5.64)	4.93 (1.28)	3.65 (0.97)	3.10 (1.02)	2.73 (0.39)	35.62 (6.81)	19.95 (4.84)
Sports		1,445 (100%)	7.91 (5.14)	7.42 (5.14)	5.16 (1.12)	3.56 (0.91)	3.36 (0.98)	2.75 (0.40)	35.43 (5.93)	19.32 (4.89)
	Yes	562 (38.9%)	7.57 (5.14)	7.34 (5.14)	5.28 (1.05)	3.71 (0.86)	3.53 (0.94)	2.77 (0.40)	35.62 (5.80)	18.79 (4.93)
	No	883 (61.1%)	8.12 (5.12)	7.47 (5.15)	5.08 (1.15)	3.47 (0.93)	3.26 (0.99)	2.74 (0.40)	35.31 (6.02)	19.65 (4.83)
Home		1,445 (100%)	7.91 (5.14)	7.42 (5.14)	5.16 (1.12)	3.56 (0.91)	3.36 (0.98)	2.75 (0.40)	35.43 (5.93)	19.32 (4.89)
	Rural area	387 (26.8%)	7.76 (5.16)	7.36 (5.18)	5.09 (1.11)	3.60 (0.91)	3.29 (1.00)	2.75 (0.40)	35.44 (5.91)	19.76 (4.94)
	Small town	611 (42.3%)	8.10 (5.19)	7.44 (5.07)	5.19 (1.12)	3.57 (0.91)	3.34 (0.98)	2.75 (0.38)	35.23 (6.06)	19.66 (4.84)
	Big town	254 (17.6%)	7.53 (4.89)	7.54 (5.34)	5.17 (1.13)	3.55 (0.92)	3.49 (0.95)	2.74 (0.45)	35.60 (5.63)	18.61 (4.72)
	City	193 (13.4%)	8.09 (5.23)	7.28 (5.06)	5.17 (1.11)	3.48 (0.92)	3.44 (0.95)	2.74 (0.42)	35.80 (5.98)	18.25 (4.92)

### 3.2 Global network

**Figure 1:**
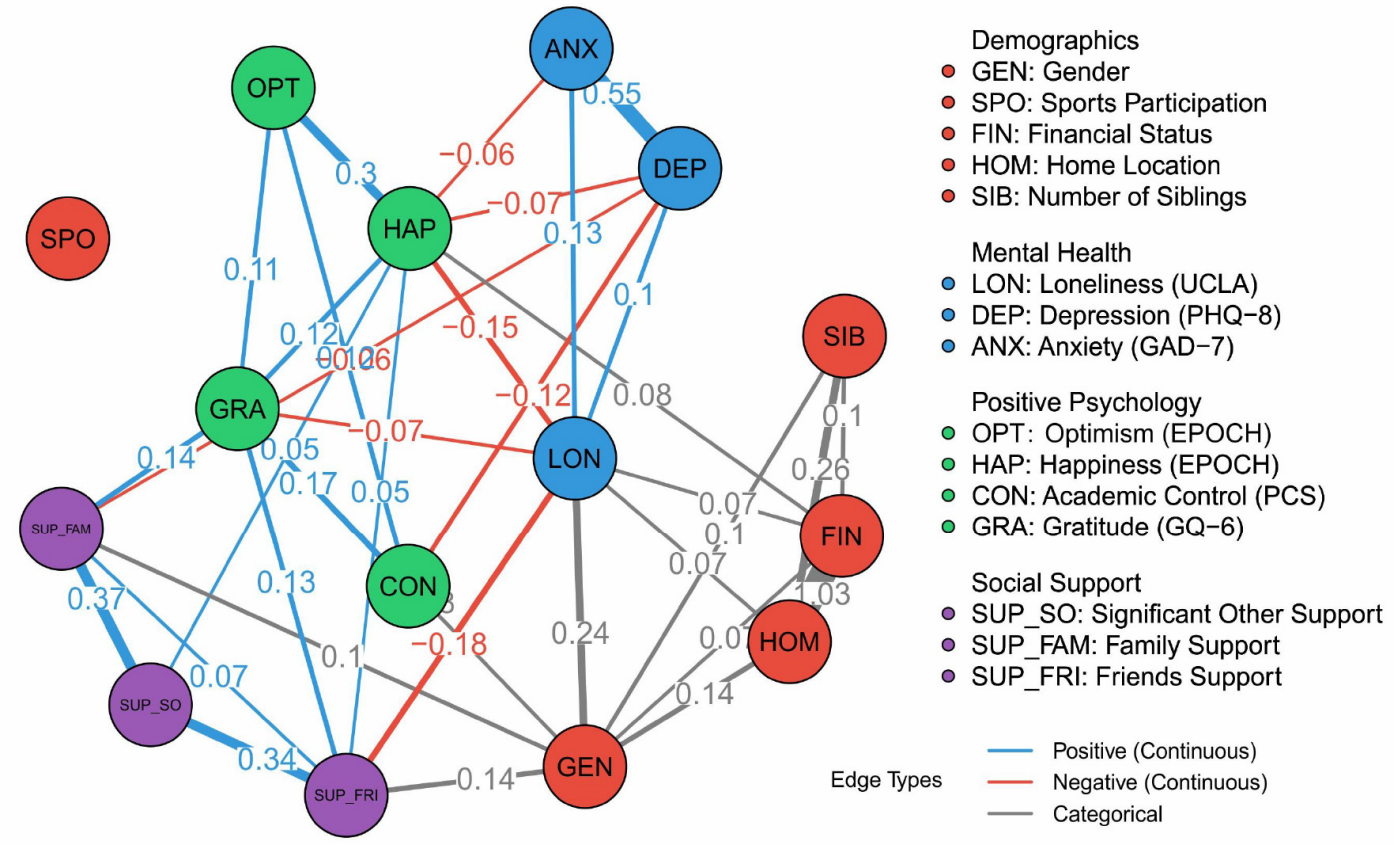
Mixed graphical model of adolescent loneliness and associated variables. **Edge thickness** indicates association strength (elastic net coefficients), with **colors** denoting direction and variable type (continuous/categorical).

The estimated global network model is presented in Figure 1, providing a comprehensive overview of the interrelations among adolescent mental health, positive psychological characteristics, social support, and key demographic factors.

The network reveals several noteworthy patterns. Depression (DEP) and anxiety (ANX) display a strong positive association (edge weight = 0.55), mirroring findings from prior research and underscoring their close co-occurrence in adolescent populations. Loneliness (LON) occupies a central position, directly linked to both higher depression and lower levels of happiness (HAP; edge weight = −0.15) and gratitude (GRA; −0.12), highlighting its pivotal role at the intersection of psychological distress and protective factors. The cluster of positive psychological variables—including optimism (OPT), happiness, gratitude, and perceived academic control (CON)—forms a densely interconnected subnetwork, with generally positive associations among themselves and inverse associations with mental health symptoms and loneliness.

The social support domain, represented by support from family (SUP_FAM), friends (SUP_FRI), and significant others (SUP_SO), is tightly interconnected (e.g., SUP_FAM–SUP_FRI: 0.34), and shows positive associations with positive psychology constructs, while buffering against loneliness.

Demographic variables show a distinct pattern. The association between financial status (FIN) and home location (HOM) is particularly strong (edge weight = 1.03), reflecting the close link between region and socioeconomic status within this sample. However, it is important to note that both FIN and HOM are categorical variables with limited categories; thus, their edge weights reflect changes in regression coefficients rather than conventional correlations, and may appear larger in magnitude than those between continuous variables. These weights are not correlation coefficients, but represent the strength of associations estimated via elastic net regression, and should be interpreted in this context.

Among demographic variables, gender (GEN) shows several modest associations with psychological and social variables, indicating that gender differences permeate multiple domains of adolescent wellbeing. Other demographic links are mostly contained within the demographic subnetwork, with weaker ties to psychological constructs.

Overall, this network analysis offers a panoramic view of the adolescent psychosocial landscape. It highlights the centrality of loneliness as a bridge between distress and resilience factors, the strong coupling of depression and anxiety, and the prominent role of positive psychology and social support as mutually reinforcing protective factors. The global model thus provides a foundation for more granular exploration of subgroup differences and key intervention targets.

**Figure 2:**
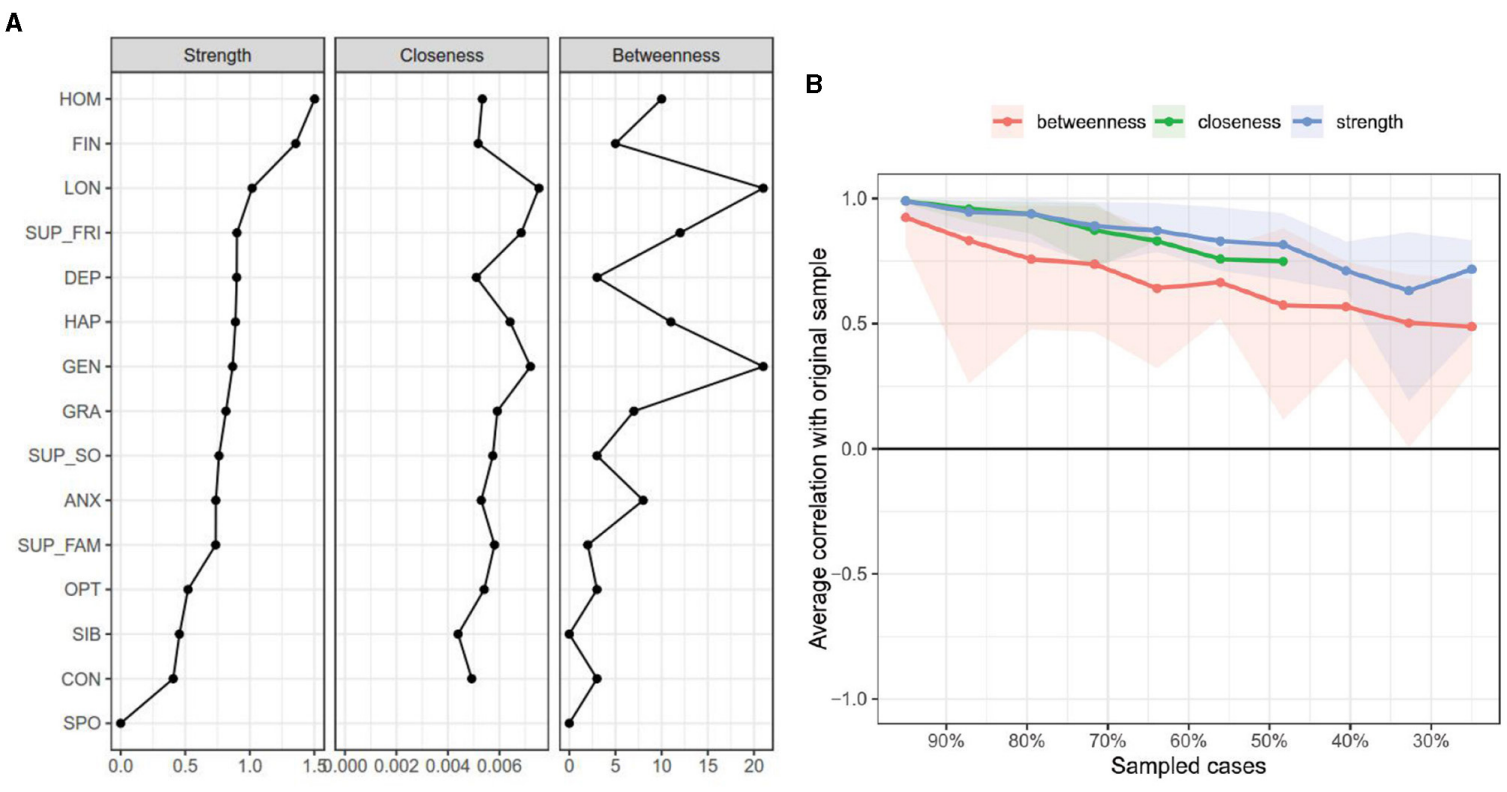
**(A)** Centrality indices of network nodes. **(B)** Stability of centrality indices as assessed by case-dropping bootstrapping.

Centrality analyses were conducted to identify the most influential nodes within the global network (Figure 2A). The results indicated that the categorical demographic variables, home location (HOM) and financial status (FIN), exhibited the highest strength centrality. However, this finding should be interpreted with significant caution. As has been noted in network analysis literature, centrality indices for categorical variables in Mixed Graphical Models are not directly comparable to those for continuous variables and can be artificially inflated. This inflation occurs because the edges connected to categorical variables represent regression coefficients, not correlations, and their magnitude is not bounded between −1 and 1. When a categorical variable with few levels is strongly predictive of another variable—as is the case with the tight link between home location and financial status in our sample—the corresponding regression coefficient can be very large, disproportionately increasing its strength centrality. Therefore, the interpretations should be cautious. Stability results, as depicted in Figure 2B, are critical for guiding this interpretation. The strength and expected influence centrality indices were found to be moderately robust, with CS-coefficients of 0.439 and 0.517, respectively. As these values are above the recommended threshold for interpretability, we consider these indices reliable. In contrast, closeness and betweenness centrality exhibited very low stability (CS-coefficients near zero). Consequently, these metrics are unreliable for this dataset and will not be interpreted. Our discussion of centrality will therefore be restricted to the stable indices. Among the psychological constructs, loneliness (LON) consistently exhibited the highest strength centrality, underscoring its robust and direct connections to other key variables in the network. This identifies it as the most influential psychological node in terms of its overall connectivity.

### 3.3 Network analysis by sex

**Figure 3:**
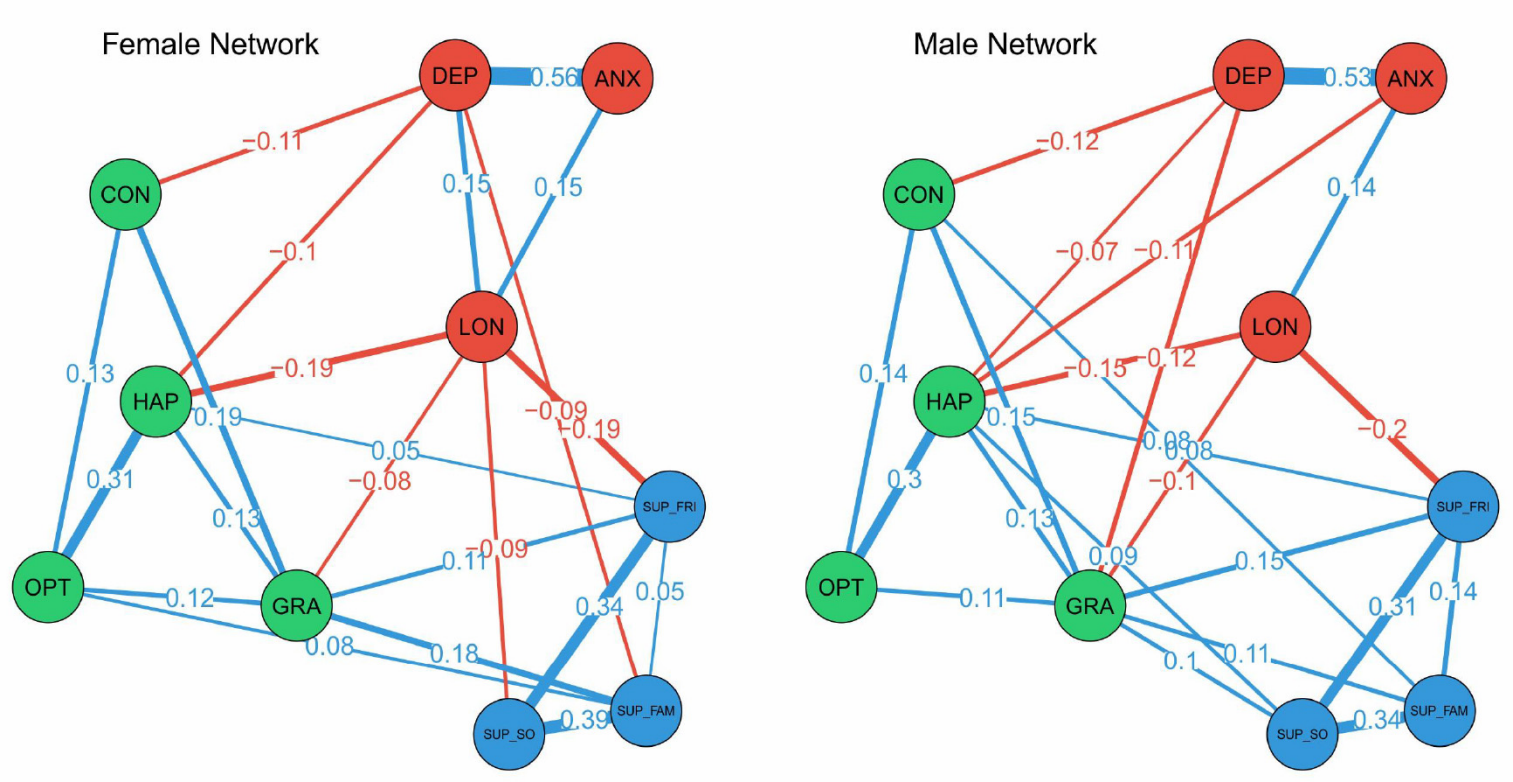
Gaussian graphical model networks by sex. **Edge thickness** reflects association strength; positive and negative associations are denoted in **blue** and **red**, respectively.

The network analysis revealed several gender-specific differences in the pattern of associations (see Figure 3). Notably, in the female network, there was a positive connection between depression and loneliness (0.15), which was not significant appeared in the male network. The negative association between loneliness and happiness was also stronger for females (−0.19) than males (−0.15), suggesting a tighter link between subjective wellbeing and loneliness among girls. Conversely, the negative association between loneliness and support from friends was considerably stronger for males (−0.20) than females (−0.09), indicating a more pronounced buffering role of peer support for boys. Additionally, distinct connections emerged only in the male network. For males, there was a negative association between happiness and anxiety (−0.11), as well as a negative association between gratitude and depression (−0.07); these links were not significant appeared in the female network. These findings highlight that certain pathways—particularly those linking depression, happiness, and gratitude with other psychosocial factors—differ by gender, and may inform the development of tailored strategies for improving adolescent wellbeing.

**Figure 4:**
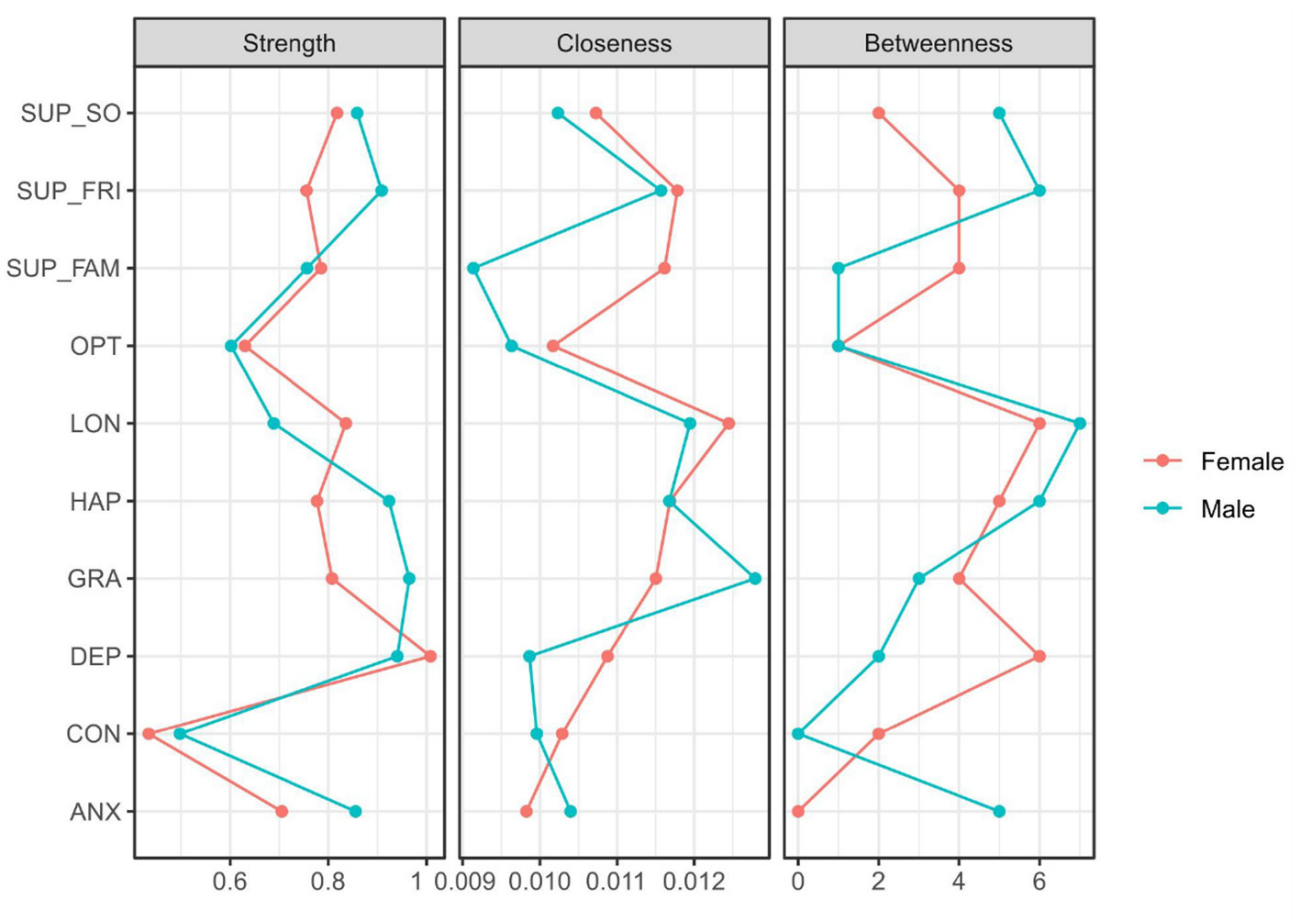
Centrality indices by sex; **red** for females, **blue** for males.

Centrality analysis based on the stable strength index revealed distinct gender patterns in the roles of support and mental health variables (see Figure 4). For males, support from friends (SUP_FRI) showed higher strength centrality, underscoring the pivotal role of peer relationships in their psychosocial network. In contrast, family support (SUP_FAM) was more central for girls. Additionally, depression (DEP) exhibited higher strength centrality in the female network, whereas anxiety (ANX) was more central among males. These findings suggest that based on the overall strength of connections, girls' psychosocial health may be more influenced by family dynamics and depressive symptoms, while boys' networks are more influenced by anxiety and anchored more strongly by peer support.

To formally test for gender differences, a permutation-based Network Comparison Test (NCT) was conducted. The results revealed no significant difference in the overall network structure (*p* = 0.39) or global strength (*p* = 0.73) between the male and female networks. This suggests that, at a global level, the psychosocial networks of boys and girls are largely similar in their overall connectivity. However, given our *a priori* interest in gender-specific pathways, we proceeded with exploratory, *post-hoc* comparisons of individual edges. It is important to interpret these findings with caution, as they are exploratory in nature. The tests identified several specific edges that differed significantly between genders. Notably, the positive association between depression and loneliness was significantly stronger for females (*p* = 0.040), as was the negative association between loneliness and optimism (*p* = 0.020). Furthermore, the connections between family support and optimism (*p* = 0.040) and between depression and gratitude (*p* = 0.040) also showed significant gender differences. These findings, while preliminary, point toward specific pathways that may function differently for adolescent boys and girls.

## 4 Discussion

The present study set out to elucidate the centrality and network role of loneliness in adolescent mental health, to map the multivariate associations among psychological distress, social support, positive psychological characteristics, and demographic factors, and to systematically examine gender differences in these network structures among Kenyan secondary school students. Consistent with our hypotheses, we found that loneliness was a highly central node within the psychosocial network, characterized by strong direct associations with depressive symptoms, reduced wellbeing (happiness, gratitude), and social support. Furthermore, gender-specific network analyses revealed that key associative pathways involving loneliness and other psychosocial variables diverged notably between girls and boys. These findings underscore the pivotal, gender-contingent role of loneliness in the context of adolescent psychological health in this population.

Our global network analysis revealed that loneliness occupied the most central position among all continuous psychological variables, demonstrating robust direct connections with depression, happiness, gratitude, and social support. This supports and extends recent network-based research, which highlights loneliness as a key node structurally positioned between clusters of affective distress and protective resources in youth across diverse cultures. Notably, our findings provide direct empirical evidence from sub-Saharan Africa—a region critically underrepresented in the loneliness literature. The centrality of loneliness suggests that interventions targeting loneliness could have broad, network-wide relevance for adolescent mental health, supporting calls for loneliness prevention as a public health priority (19).

Our results show that loneliness is not only strongly associated with depressive and anxiety symptoms but is also inversely correlated with positive psychological resources such as happiness, gratitude, and optimism. This aligns with recent findings from global samples, which show that loneliness is negatively associated with both hedonic wellbeing and eudaimonic resources (20). The observed negative associations between loneliness and gratitude/happiness echo longitudinal results suggesting that social disconnection is linked to an erosion of positive affect and self-regulatory capacities in youth (21). Importantly, our work extends this literature to the Kenyan context, where protective factors such as gratitude and optimism may be especially relevant for resilience against social and economic adversity (22).

Consistent with theory, social support from family, friends, and significant others was tightly interconnected and broadly negatively associated with loneliness and psychological distress (23). However, our gender-stratified network analyses revealed important nuances. For girls, family support played a more central role (24), and their heightened vulnerability to social and emotional disconnection was further highlighted by a stronger link between loneliness and happiness, echoing broader global trends (25). Furthermore, our exploratory analysis showed the association between depression and loneliness was significantly stronger among girls. This may reflect traditional gender socialization patterns prevalent in many Kenyan communities, where girls are often expected to maintain strong ties to the family unit and take on more domestic responsibilities, making the family their primary sphere of social interaction and support. Conversely, for boys, peer support (support from friends) had a more pronounced negative association with loneliness, suggesting it serves as a more critical buffer. This finding aligns with cultural expectations that often encourage boys to develop autonomy and social competence within peer groups outside the immediate family structure, making peer acceptance particularly influential for their wellbeing. Unexpectedly, certain positive psychological variables (e.g., gratitude) also displayed unique links to distress in boys but not girls. Taken together, these findings parallel recent studies emphasizing that pathways of psychological distress and resilience are often gender-contingent (26), highlighting the need to account for these specific patterns of risk and protection.

These distinct gendered patterns offer a clear direction for designing more effective mental health interventions. Given that family support is more central for girls and their depression is more tightly linked to loneliness, programs could focus on strengthening family ties through psychoeducation for parents or communication training. Conversely, for boys who rely more on peer support to buffer loneliness, interventions leveraging peer networks—such as structured mentoring or facilitated group activities—would likely be more effective. This tailored approach allows for a more precise strengthening of the most influential support system for each gender, a particularly urgent need in LMIC contexts where resources are scarce and gender norms may be related to help-seeking and socialization.

A notable methodological observation from our global network was the apparent high centrality of the categorical demographic variables, financial status and home location. As addressed in our results, this is likely a methodological artifact of the Mixed Graphical Model, where the centrality of categorical variables can be inflated and is not directly comparable to that of continuous psychological variables. Their strong interconnection primarily reflects the underlying socioeconomic clustering within the sample—that is, the close link between where students live and their family's financial status—rather than a direct, pervasive influence on all individual psychological states. Therefore, our interpretation rightly focuses on the more robust and theoretically meaningful centrality of the psychological constructs. In contrast, gender's role as a structurally important node was more psychologically informative. This finding aligns with the persistent gender disparities in mental health observed across both high- and low-resource settings and provided a strong rationale for our subsequent gender-stratified analyses (27).

This study is subject to several limitations. The cross-sectional design precludes causal inference, as the network edges represent statistical associations rather than directional causal pathways. The generalizability of the findings is also constrained. While the sample is large and diverse within the Kenyan context, its applicability to other regions or countries may be limited. This issue of generalizability is underscored by our attrition analysis, which revealed that our final cohort reported significantly higher levels of depression and anxiety (small effects) and, most notably, loneliness (a medium effect) compared to excluded participants. This suggests a potential selection bias, wherein adolescents experiencing greater distress were more likely to complete the survey. It is plausible that this over-representation of distressed individuals may have amplified the strength of the associations between loneliness and negative affective states, thereby inflating the centrality of loneliness in our network model. Therefore, the findings should be interpreted with caution, as they may be most representative of a help-seeking or more psychologically distressed subgroup of adolescents. On a methodological note, the analysis of gender differences in specific network edges should be considered exploratory, as these *post-hoc* comparisons were intended to be hypothesis-generating. Finally, it is important to acknowledge that the network approach itself, while powerful for mapping associations, may not capture latent constructs or unmeasured confounders (28).

## 5 Conclusion

This study demonstrates that loneliness occupies a highly central position within the psychosocial networks of Kenyan adolescents, acting as a key bridge between psychological distress and protective factors such as social support, optimism, and gratitude. Gender-specific analyses reveal that the pathways linking loneliness with other mental health and wellbeing variables differ notably between boys and girls, underscoring the importance of considering gender when designing interventions. These findings highlight the urgent need to address loneliness as a core target in adolescent mental health strategies in sub-Saharan Africa, and suggest that network-based approaches offer valuable insights for identifying effective and contextually appropriate intervention points.

## Data Availability

Publicly available datasets were analyzed in this study. This data can be found here: https://www.sciencedirect.com/science/article/pii/S2352340923002007.
